# *Sirepo*: an open-source cloud-based software interface for X-ray source and optics simulations

**DOI:** 10.1107/S1600577518010986

**Published:** 2018-10-19

**Authors:** Maksim S. Rakitin, Paul Moeller, Robert Nagler, Boaz Nash, David L. Bruhwiler, Dmitry Smalyuk, Mikhail Zhernenkov, Oleg Chubar

**Affiliations:** a NSLS-II, Brookhaven National Laboratory, Upton, NY, USA; b RadiaSoft LLC, Boulder, CO, USA; c Bivio Software Inc., Boulder, CO, USA; d Earl L. Vandermeulen High School, Port Jefferson, NY, USA

**Keywords:** *Synchrotron Radiation Workshop* (*SRW*), *Sirepo*, X-ray optics simulations, cloud-based software

## Abstract

*Sirepo*, a browser-based GUI for X-ray source and optics simulations, is presented. Such calculations can be performed using *SRW* (*Synchrotron Radiation Workshop*), which is a physical optics computer code, allowing simulation of entire experimental beamlines using the concept of a ‘virtual beamline’ with accurate treatment of synchrotron radiation generation and propagation through the X-ray optical system. *SRW* is interfaced with *Sirepo* by means of a Python application programming interface. *Sirepo* supports most of the optical elements currently used at beamlines, including recent developments in *SRW*. In particular, support is provided for the simulation of state-of-the-art X-ray beamlines, exploiting the high coherence and brightness of modern light source facilities. New scientific visualization and reporting capabilities have been recently implemented within *Sirepo*, as well as automatic determination of electron beam and undulator parameters. Publicly available community databases can be dynamically queried for error-free access to material characteristics. These computational tools can be used for the development and commissioning of new X-ray beamlines and for testing feasibility and optimization of experiments. The same interface can guide simulation on a local computer, a remote server or a high-performance cluster. *Sirepo* is available online and also within the NSLS-II firewall, with a growing number of users at other light source facilities. Our open source code is available on GitHub.

## Abstract 2

*Sirepo*, an open-source browser-based interactive GUI for X-ray source and optics simulations, performed by *SRW* (*Synchrotron Radiation Workshop*), is presented.

## 1. Introduction   

We present an easily accessible and convenient approach to performing scientific simulations without the need of multi-platform installation and maintenance. Our novel framework, *Sirepo*, provides the combination of a browser interface with a back-end server able to serve many different software packages. We discuss in detail a recently implemented interface with *SRW*, a high-accuracy general physical optics computer code for synchrotron radiation (SR) source and X-ray optics calculations and simulation of experiments (Chubar & Elleaume, 1998 ▸; Chubar *et al.*, 2002 ▸, 2013*a* ▸), which was previously integrated with the Igor Pro package (https://www.wavemetrics.com) and Python.

There are other X-ray optics codes and interactive frameworks in the field. *URGENT* (Walker & Diviacco, 1992 ▸) is a computer program for calculating undulator radiation with spectral, angular, polarization and power density characterization. *SPECTRA* (Tanaka & Kitamura, 2001 ▸) is a SR calculation code. *WavePropaGator* (*WPG*) (Samoylova *et al.*, 2016 ▸) is an interactive framework for X-ray free-electron laser optics design and simulations. *SHADOW3* (Sanchez del Rio *et al.*, 2011 ▸) is an open-source ray-tracing code for modeling optical systems. *ShadowOui* (Rebuffi & Sánchez del Río, 2016 ▸) is a visual environment for X-ray optics and synchrotron beamline simulations. *Orange Synchrotron Suite* (*OASYS*) (Rebuffi & Sanchez del Rio, 2017 ▸) is an open-source graphical environment for optics simulation software used at synchrotron facilities, based on *Orange 3* (https://orange.biolab.si) workflow software.

In addition to *SRW*, *Sirepo* supports simulations with the following codes: *SHADOW3* is mentioned above; *Elegant* is a code for simulation of charged-particle accelerators (Borland, 2000 ▸); *Warp* is used for simulation of high-intensity charged-particle beams and plasmas in both the electrostatic and electrodynamic regimes (Grote *et al.*, 2005 ▸; Friedman *et al.*, 2014 ▸); *Hellweg* simulates traveling wave electron linacs with beam loading (Kutsaev, 2010 ▸). The source code of *Sirepo* could be extended to support codes from different scientific domains such as condensed matter physics and material science, chemistry and biology, and other areas making use of codes requiring complex and at the same time flexible input and comprehensive output in the form of interactive visualization, raw data files and unified exchange format.

The *Sirepo* interface to *SRW* provides the advantages of the Python and Igor Pro interfaces while avoiding their limitations, resulting in an open-source multi-user browser-based GUI with the *SRW*–Python interface on the server-side. This enables portable and reproducible simulations with a minimum of installation and configuration. Key advantages of a browser-based GUI and cloud computing include immediate access to computational resources, sharing information for collaborative work, *etc*. The publicly available server (https://sirepo.com) allows users to start using *SRW* (and other codes) immediately without a local installation.

This paper focuses on the browser interface *Sirepo* which is built on top of the *SRW* code. The latter code is being developed independently; however, the authors make efforts to make both codes as compatible as possible. The existing versions of the codes are still evolving, and the code and documentation will be updated in the future.

## 2. Software environment   

*Sirepo* is a distributed system, which involves a front-end, *i.e.* a JavaScript client accessible *via* a browser, and a back-end service, running on an isolated server computer or on a cluster. A typical workflow is as follows: a user prepares a simulation in a browser using the JavaScript GUI on a desktop computer/laptop or even a mobile device, then an HTTP(s) request is sent *via* network to the *Sirepo* back-end server. The server then processes the request and executes corresponding simulations within a computing environment, and finally the calculation result is returned *via* network to the same user’s interface in the browser and is visualized in the form of interactive plots with corresponding data files. We discuss all of these steps and their implementation.

### 2.1. *SRW* library   

*SRW* provides unique capabilities to simulate a whole SR beamline starting from source to a sample/detector position, and even entirely simulate some types of experiments making use of the high brightness and coherence of modern SR sources. The code is able to generate data on spectral, spatial and polarization characteristics of the radiation in the near-field and far-field approximations produced by a relativistic electron beam traveling in external magnetic fields of arbitrary configuration. It uses rapid numerical algorithms for different types of SR, including bending-magnet radiation (from central parts and edges), undulator and wiggler radiation. For these calculations the modeled or measured magnetic field can be used. The computation can take into account electron beam emittance and energy spread.

*SRW* is used at many light source facilities to calculate the spectral performance of insertion devices, taking into account details of their magnetic design and, if necessary, magnetic field errors. It is used for high-accuracy physical-optics-based simulation of SR propagation through optical elements, taking into account their eventual imperfections, and optimization of entire optical schemes of beamlines dedicated for infrared, UV and X-ray spectral ranges. *SRW* is also used for the simulation of some types of experiments with SR, electron beam diagnostics, optical element quality characterization and other applications.

The *SRW* core code is written in C++. The initial version of the code was interfaced with Igor Pro (WaveMetrics) and recent versions also include a Python interface. Various simulations of a beamline can be carried out either using the basic Python interface or a dedicated ‘virtual beamline’ application programming interface (API). The Python version of *SRW* supports sequential and parallel simulations. There are three main types of parallelization used in *SRW*:

(i) Message passing interface (MPI) parallelization implemented *via* the Python bindings for the MPI libraries in the 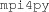 Python package (https://mpi4py.readthedocs.io). This enables efficient parallelization of computations on isolated servers and high-performance computer clusters.

(ii) Open multi-processing (OpenMP) parallelization is used for time-dependent simulations for XFEL applications (https://github.com/SergeyYakubov/SRW/tree/openmp). OpenMP is used for parallelization within one multi-core server, using the shared memory multi-threading approach, while MPI is used for parallelization between computational nodes.

(iii) Open computing language (OpenCL) parallelization, utilizing graphics processing units (GPUs), was tested on an example of the SR calculation in collaboration with the Canadian Light Source. This work is still in progress.

One of these parallelization methods can be used at a time. We also note that some parts of the OpenMP and OpenCL parallelization implementation of *SRW* will be incorporated into the main *SRW* source code in the future.

### 2.2. Server-side implementation   

The server-side (back-end) of *Sirepo* is based on *Flask* (http://flask.pocoo.org), which is a lightweight framework for web development with Python (https://www.python.org). *Flask* depends on the Werkzeug toolkit, a Python utility library for the Hypertext Transfer Protocol (HTTP) and Web Server Gateway Interface (WSGI), allowing fast development of high-quality secure web applications. In our production environment, we use an industry-standard Nginx HTTP(s) server as a reverse proxy. It allows scalable and reliable solutions to be built thanks to its event-driven (asynchronous) architecture.

To exchange data between the server and clients we use JavaScript Object Notation (JSON), a lightweight data-interchange format supported by many programming languages. Since *Sirepo* is intended to be used by many scientists within a single facility or around the World, it is critical to have a reliable job management system. For that purpose, we use Celery (http://www.celeryproject.org) and RabbitMQ (https://www.rabbitmq.com). Celery provides an asynchronous job queue for executing long-running simulations and provides cluster management. Celery uses RabbitMQ as a message broker, which provides the communication between the web server and the cluster of back-end execution servers.

Parallel execution of CPU-intensive calculations with *SRW* and other codes is implemented by means of MPI, which is the technology used to run scientific codes in parallel across a cluster of computational nodes on a network. For quality assurance, we use the Pytest (https://docs.pytest.org) framework allowing for exercising and testing individual features of the Python implementation.

### 2.3. Client side implementation   

The *Sirepo* client (front-end) is based on Hypertext Markup Language (HTML5) which is a language used for structuring and presenting web content. It involves JavaScript, Cascading Style Sheets (CSS3), Scalable Vector Graphics (SVG) and other libraries. CSS3 is a style sheet language used for describing the presentation of a document written in a markup language. For more consistent rendering, we use Bootstrap, an HTML, CSS3 and JavaScript framework for developing cross-platform web applications. We also use AngularJS, a structural framework for dynamic web applications. For data visualization, we use the D3 (http://d3js.org) JavaScript graphics library, which is used to generate interactive plots in a browser. D3 supports large datasets with dynamic behavior, proving to be both powerful and relatively easy to integrate into the AngularJS framework.

In *Sirepo*, visual data are represented as so-called ‘reports’, which can show interactive one-dimensional (1D) and two-dimensional (2D) plots. These reports have menus, where parameters of various calculation types can be controlled. The reports can also export data in the form of images with specified resolution, or the corresponding raw data, as well as the Python source code used to perform the calculation.

We implemented support for ‘heatmaps’ (*i.e.* 2D image plots) of intensity distributions and other data. The mouse or keyboard or touchscreen can be used to interactively zoom and pan the plot for better detail. Zooming could be performed for both axes simultaneously (if the cursor hovers over the heatmap area) or independently (if the cursor hovers over a corresponding 1D ‘cut’ below or on the right). The line plots allow the user to find the coordinates of a specific point, which can be useful for finding minima and maxima as well as any intermediate values of functions that are plotted. With the cursor hovering over a line, a mouse click causes the coordinates of the closest local maximum to appear in the widget, together with (if applicable) the full width at half-maximum (FWHM), a frequently used quantity to estimate the beam size, the width of a harmonic, *etc.* The location of the marker can be changed *via* the keyboard’s left/right and up/down buttons. The range of the plot can be dynamically changed using the scroll wheel of a mouse. In the following sections, these and other functions will be illustrated.

### 2.4. *Sirepo* interface details   

*Sirepo* can be used in any modern browser, with *SRW* running on a remote server or a cluster, including cloud-based dynamically allocated virtual machines (VMs). With this modern interactive JavaScript GUI, expert users can at any time export a valid Python script for an individual simulation/report for further specialization and command-line execution. Cloud-based *SRW* is actively used at the publicly available website https://sirepo.com. Two high-performance servers with *Sirepo*/*SRW* installations are available and routinely used at National Synchrotron Light Source (NSLS-II) at Brookhaven National Laboratory (Rakitin *et al.*, 2017 ▸) by users and scientists on the campus network.

**Figure 1:**
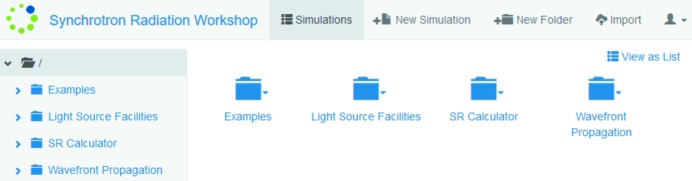
Simulations page of *Sirepo*.

**Figure 2:**
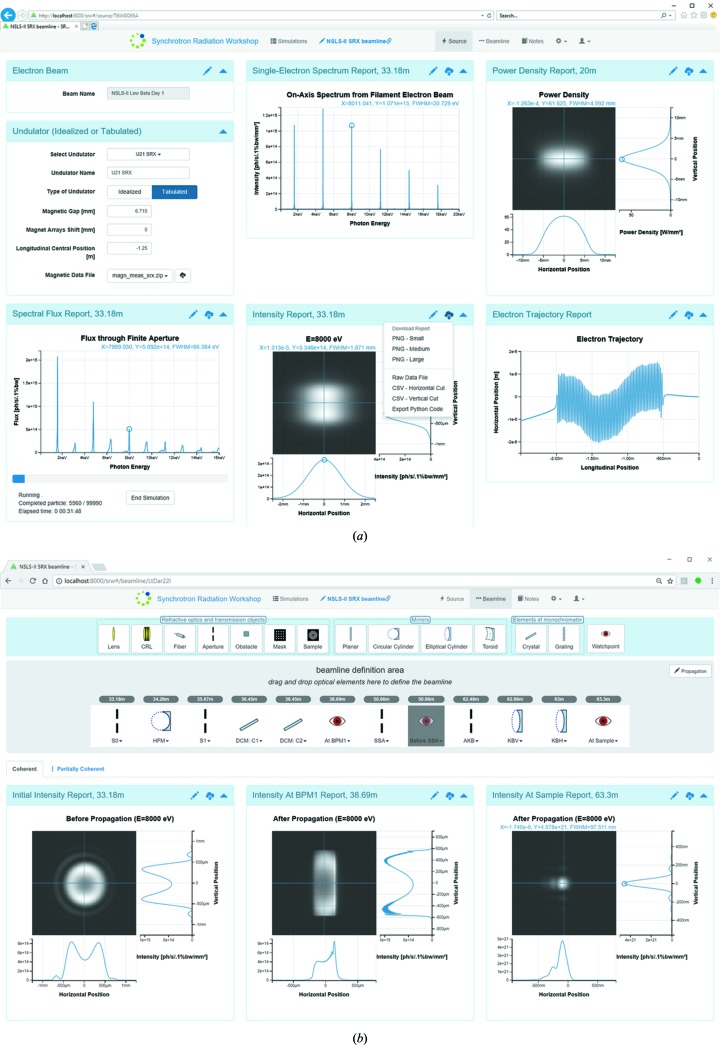
Typical Source (*a*) and Beamline (*b*) pages of *Sirepo*.

Both single-electron (one-pass and usually fast) and multi-electron (often long-running) simulations have been implemented. A special manager allows users to organize simulations, *i.e.* create new ones from scratch, copy an existing one, delete, rename and group different simulations in folders, as illustrated in Fig. 1 ▸. Once a simulation is selected, a user can enter either the corresponding Source or the Beamline page, which will be discussed in detail in the next section (see Fig. 2 ▸).

Authentication on *Sirepo* servers is implemented by means of the OAuth 2.0 authorization framework, and *Sirepo* currently accepts GitHub (https://github.com) logins for users. This feature enables a persistent workspace, storing all of user’s simulations under one account, no matter which browser or device are used for their creation. In ‘expert’ mode, users can see the ‘user’ icon in the upper-right corner of the interface (see Fig. 1 ▸), which allows users to authenticate themselves.

Fig. 2 ▸ displays commonly used controls of the various ‘reports’. Detailed parameters of the reports can be found by clicking on the ‘pencil’ edit icon. The parameters for each report are logically and intuitively grouped for users’ convenience: basic frequently changed parameters are usually placed on the ‘Main’ tab, while other more specific parameters are placed in separate tabs. The reports rerun automatically when the user clicks the ‘Save Changes’ button. The parameters menu of each report has the ‘question mark’ button which opens a new browser tab, showing the associated online Wiki page at GitHub; https://github.com/radiasoft/sirepo/wiki) with a detailed explanation of a particular report, where applicable. Users have the capability to download a raw data file and images in the report in three different resolutions by means of the ‘Cloud-Download’ button. Users can also minimize individual reports by clicking on the ‘triangle’ icon in the upper-right corner; in this case, the corresponding calculation for the report will not be performed, speeding up subsequent simulations.

**Figure 3:**
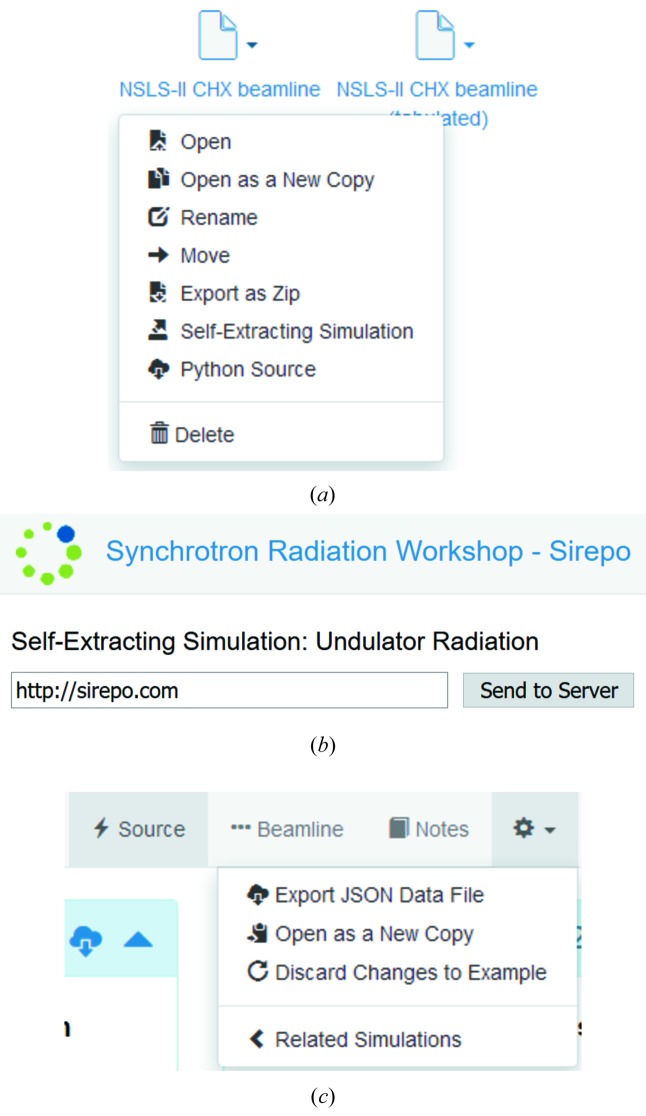
Export options in *Sirepo*: (*a*) the drop-down menu in the simulations list showing export options of a zip-archive, self-extracting simulation, and Python source code, (*b*) appearance of the exported self-extracting simulation dialog and (*c*) the ‘cog wheel’ menu showing export option of a JSON data file.

In many cases, *SRW* simulations involve additional files, *e.g.* magnetic measurement data for a tabulated undulator, or mirror surface height profile errors. Such files are either pre-defined in *Sirepo* or can be uploaded by a user. In that case a simulation cannot be exported as a single Python file. For that purpose, we implemented exporting of a zip-archive with Python, JSON and all related data files [Fig. 3(*a*) ▸], ‘Export as Zip’ menu item). Also, an advanced exporting feature of a self-extracting simulation in HTML format was implemented [Fig. 3(*a*) ▸, ‘Self-Extracting Simulation’ menu item] to allow a one-click importing to a remote server [Fig. 3(*b*) ▸].

The same menu provides a way to export the Python script used for execution of the simulation corresponding to the report [Fig. 2(*a*) ▸, see drop-down menu in the Intensity Report, ‘Export Python Code’ menu item]. This allows the end user to run an *SRW* simulation from the command line and extend the simulation script with capabilities not yet supported by the GUI. Hence, the expert user is benefited by the GUI just as much as the novice user, and the GUI never inhibits or limits what an X-ray scientist can do with *SRW*. As an accompanying tool, we have implemented exporting a single JSON file, which fully defines the simulation [Fig. 3(*c*) ▸, ‘Export JSON Data File’ menu item). This assures portability of *Sirepo* simulations.

To provide a robust mechanism for sharing the simulations across multiple installations of *Sirepo* and *SRW*, we complemented the exporting capabilities with advanced import features. The Wiki pages describe in detail the process of sharing (backing up) a simulation (https://github.com/radiasoft/sirepo/wiki). *Sirepo* accepts import of correctly formatted ‘Virtual Beamline’ Python scripts, as well as previously exported JSON files.

When a user imports a Python script, optional command-line arguments can be provided (for instance, to select the desired beamline layout to import). Import of a previously exported simulation in the form of a zip-archive can be performed in the same manner.

Magnetic measurement data for a tabulated undulator can be imported at the Source page of *Sirepo*, while the mirror surface height profile error can be uploaded at the Beamline page.

Below we explain a typical workflow for simulations using *Sirepo* and *SRW*.

## 3. SR calculations   

The following types of *SRW* calculations are supported in *Sirepo*: trajectory of electrons traveling through an arbitrary magnetic field, single-electron SR spectral flux per unit surface area, spectral flux of radiation by finite-emittance electron beam per unit surface area or within a finite aperture, SR power density distributions, fully or partially coherent radiation propagation through beamline optics. *SRW* users typically start by setting up the parameters of an SR source, which involves the parameters of the electron beam and the magnetic field source (*e.g.* an ‘idealized’ or ‘tabulated’ undulator, or a bending magnet), or a Gaussian radiation beam (*e.g.* from a free-electron laser). The next step, which is optional, consists of checking the trajectory of the relativistic electrons traveling through the magnetic field (important for insertion device sources).

Particular characteristics of interest include the resulting radiation spectrum: the single-electron spectrum, the flux through a finite aperture, as well as monochromatic intensity and power density distributions at some distance from the source. Thus, the Source page of *Sirepo* enables users to specify all the parameters of the source and perform such SR calculations, described in detail in the following sub-sections. Alternatively, users can define the source approximately as a coherent Gaussian beam.

### 3.1. Electron beam parameters   

**Figure 4:**
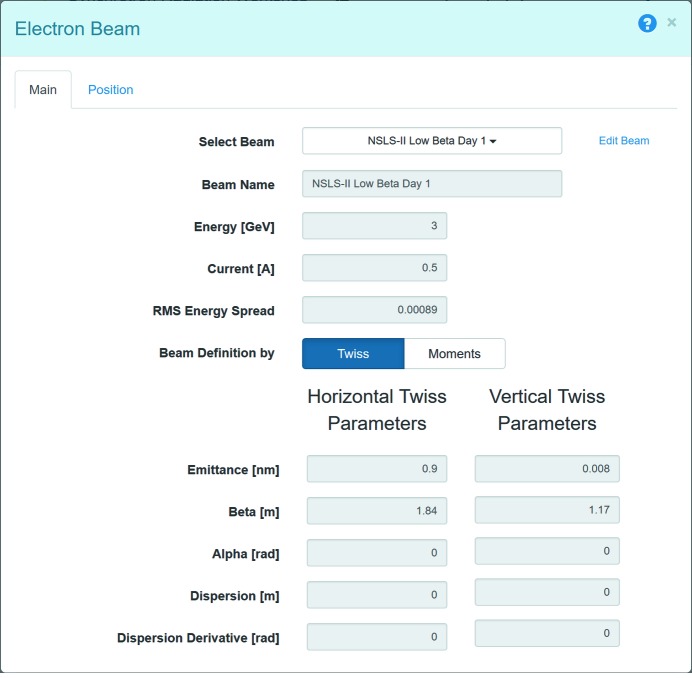
Input dialog for electron beam parameters.

To define or edit the parameters of the electron beam, users can click on the ‘pencil’ button in the heading of the Electron Beam widget at the Source page. Fig. 4 ▸ shows the menu for the parameters of the electron beam. In *Sirepo* there are a number of predefined ‘electron beams’ with nominal parameters (electron beam current, vertical and horizontal emittance, *etc.*) from existing light source facilities. Those parameters are dynamically obtained from *SRW* to ensure their correspondence to the base code. One can either use the predefined parameter values, or tune the parameters as necessary – in this case a user-defined copy of the beam will be created, which will be shared across all user’s simulations.

The electron beam is by default defined by the Twiss parameters as shown in Fig. 4 ▸, but can also be defined by the second-order statistical moments (RMS size, RMS divergence, *etc.*). The Position tab allows one to specify the longitudinal position for which the electron beam parameters (in particular, first- and second-order statistical moments) are defined. This is required for unambiguous definition of electron trajectories. The longitudinal position is calculated automatically for an idealized undulator and corresponds to a location before the undulator.

### 3.2. Undulator parameters   

**Figure 5:**
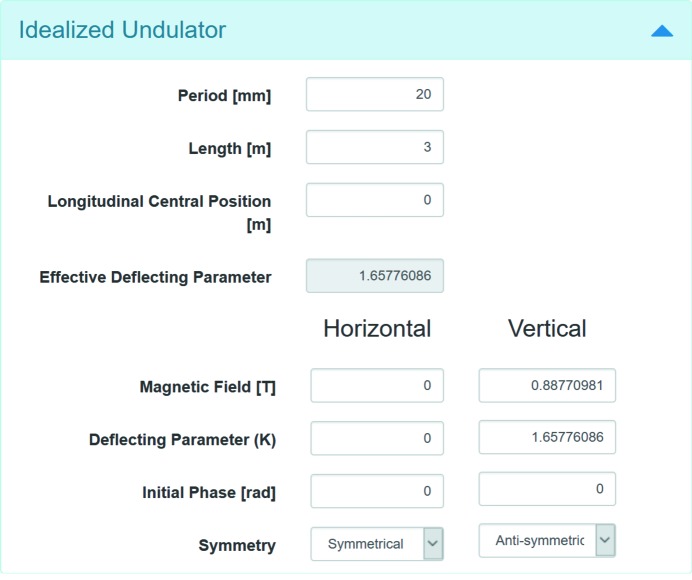
Input dialog for idealized undulator parameters.

After the parameters of the electron beam are set, one needs to define parameters of a device producing magnetic field, which is often an undulator for a modern light source facility. *SRW* supports the definition of either a sinusoidal undulator magnetic field or a tabulated field *versus* longitudinal position, which can result from magnetic simulations or measurements. The second method provides a more accurate method to simulate undulator radiation spectra and predict their realistic performance, *e.g.* at different magnetic gap values. This method is now routinely used for commissioning of NSLS-II beamlines. In agreement with *SRW*, in *Sirepo*, an undulator source can also be defined either as an ‘idealized undulator’ or a ‘tabulated undulator’. Fig. 5 ▸ shows the layout of the dialog for defining parameters of the idealized undulator while an example of a tabulated undulator is shown in Fig. 2(*a*) ▸.

In the case of the idealized undulator, users ‘tune’ the radiation spectrum by changing either the deflecting parameter *K* or the amplitude of the magnetic field (the parameters are mutually dependent and are recalculated interactively if one of them changes). In the case of the tabulated undulator, users can tune the spectrum by changing the undulator gap. In that case the magnetic field is calculated by interpolation from a set of fields tabulated for different gap values. It is possible to either use one of the predefined archives with sample magnetic measurement data in a special ASCII format or to upload a new archive in the same format.

### 3.3. Electron Trajectory Report   

The Electron Trajectory Report provides a convenient and straightforward way to verify the trajectory of electrons traveling through the magnetic field of an insertion device, which helps to characterize, for example, the quality of its shimming and to better understand the resulting radiation spectrum (see the Trajectory Report in Fig. 2*a* ▸).

### 3.4. Single-electron radiation reports: spectrum and intensity   

**Figure 6:**
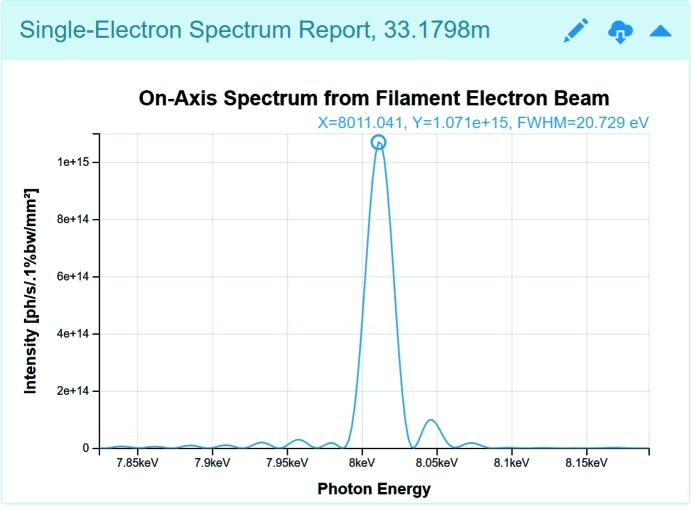
Single-Electron Spectrum Report showing the zoomed seventh harmonic of the undulator radiation spectrum presented in Fig. 2(*a*) ▸.

The Single-Electron Spectrum Report is presented in Fig. 6 ▸. The plot shows the zoomed seventh harmonic of the undulator radiation spectrum presented in Fig. 2(*a*) ▸, generated from tabulated magnetic field data. The harmonic shape is impacted by magnetic field errors.

**Figure 7:**
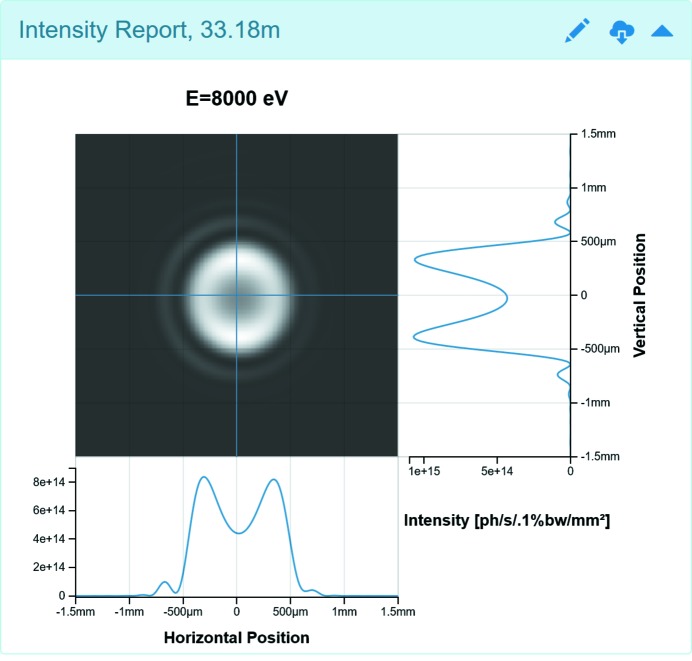
The Intensity Report from the simulation of Fig. 2(*a*) ▸, showing the single-electron intensity distribution. The corresponding report in Fig. 2(*a*) ▸ shows the multi-electron intensity, estimated by convolution from the single-electron distribution, disregarding energy spread.

The 2D image plots in *Sirepo* are used extensively for the Intensity and Power Density Reports, showing the intensity and power density distributions correspondingly at a distance from the source. Users can also see 1D cuts of the plots. Fig. 7 ▸ demonstrates the Intensity Report of the Source page, showing the single-electron intensity distribution. The Intensity Report in Fig. 2(*a*) ▸ depicts the corresponding multi-electron distribution, which was obtained from the single-electron distribution by convolution (disregarding the contribution from the electron beam energy spread). This type of fast simulation can usually give a reasonable estimate of the resulting multi-electron intensity distribution. However, a more accurate multi-electron intensity distribution, taking into account the energy spread, can also be computed using the same method that is applied for partially coherent multi-electron calculations (see §4).

As seen in Fig. 2(*a*) ▸ (on the 1D cut at the bottom of the 2D image plot of the Intensity Report) and Fig. 6 ▸, the coordinates of the marker are shown right above the plot along with the corresponding FWHM value (if it can be computed). In Fig. 6 ▸, *X* corresponds to the photon energy and *Y* corresponds to the intensity value at that particular point. The plot can be zoomed by scrolling the mouse wheel. For users’ convenience, the photon energy is displayed in the title of the plot, while the location where the intensity is observed is shown next to the name of the report.

### 3.5. Partially coherent/multi-electron calculation reports: Spectral Flux (per unit surface area)   

The Spectral Flux Report supports two types of calculations used mainly for undulator radiation. The first type is an approximate fast calculation of the spectra which ignores errors of the magnetic field. The second type is an accurate but time-consuming calculation using the method of macro-particles, that is particularly useful for realistic simulations taking into account magnetic field errors and other effects. The report is designed to perform a long-running parallel simulation involving a large number of ‘macro-electrons’, traveling in a (possibly imperfect) magnetic field of an undulator, and to accurately predict the spectral performance. The interactive report is updated periodically to visualize the most recent data from the multi-electron calculation performed by *SRW* [see the lower-left report in Fig. 2(*a*) ▸]. The progress bar shows the current progress of the calculation in terms of the number of macro-electrons used.

### 3.6. Brightness   

The Brightness Calculation Report was recently added to allow the comparison of brightness at different facilities. The classic formulae were given by Kim (1989 ▸) with correction due to electron beam energy spread elucidated by Tanaka & Kitamura (2009 ▸). We implement a variant on Tanaka’s formalism that has already been included in the Igor Pro interface to *SRW*. In addition to the inclusion of the energy spread effects on undulator radiation spectral flux, angular divergence and effective source size, the possibility of taking into account a deviation from undulator resonant frequency is also included in the calculation method implemented in *SRW* (Nash *et al.*, 2018 ▸). This is not accounted for in the results of Tanaka *et al.*, and this energy detuning is an important effect since most undulators operate at a detuned photon energy in order to maximize flux.

## 4. Radiation propagation simulations   

The calculation of radiation wavefront propagation through an optical system is very important for understanding beamline operation at a light source facility. In the *SRW* code, such calculations are performed using high-accuracy physical-optics-based methods. The central part of this method consists of propagating a fully coherent radiation wavefront through a set of optical elements, using Fourier optics. This fully coherent wavefront corresponds to emission from a single macro-electron (Chubar & Elleaume, 1998 ▸). This calculation is usually fast (lasting from seconds to minutes). The calculation of characteristics of propagated partially coherent radiation is carried out by averaging of the single-electron characteristics over the phase-space volume of the electron beam. This is usually a CPU-intensive calculation, which is executed in *SRW* using parallel processing.

The ‘Beamline’ page (Fig. 2*b* ▸) provides users with means to simulate optical beamlines. A number of utility functions and libraries were created to facilitate application of the general ‘thin element’ transmission-function-based propagator for different types of refractive and diffractive optical elements. Such elements include perfect and imperfect compound refractive lenses, zone plates, special coherence probes, masks, *etc.* This approach is also applied to miscellaneous samples for the simulation of coherent scattering experiments.

The *Sirepo* interface to *SRW* is designed to be easy to use and intuitive. Default values for electron beam, magnetic field source and beamline components are selected to give physically meaningful results. In adjusting the parameters and building up a beamline, however, the user is required to have some knowledge of the different elements and X-ray propagation in order to arrive at an accurate result. In particular, grid sizes, precision parameters and propagation parameters must be adjusted to ensure adequacy of the calculations. A number of dedicated physical optics propagators for different types of reflective optics and crystals have been developed in *SRW* recently (Sutter *et al.*, 2014 ▸; Canestrari *et al.*, 2014*a* ▸,*b* ▸). These propagators were extensively benchmarked and extended by including various options and imperfections of the optical elements. All these propagator options are available in *Sirepo* *via* dedicated menus, dialogs, widgets and Wiki-pages of documentation. Some automation is available for different methods of wavefront propagation. The propagation parameters may be adjusted in a convenient dialog, but some care is required in their settings. These parameters are documented in the *Sirepo* Wiki (https://github.com/radiasoft/sirepo/wiki/SRW-Propagation-Parameters ) along with each of the beamline elements and reports. This documentation is under development, and will continue to improve. We also foresee improved automation of parameter setting, although some expertise in this area will likely remain necessary.

**Figure 8:**
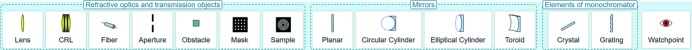
Optical elements available on the Beamline page of *Sirepo*.

Fig. 8 ▸ depicts all optical elements available in *Sirepo* at the time of this writing. The optical elements are organized in the following groups: *refractive optics and transmission objects* [lens, compound refractive lens, fiber, aperture, obstacle, ‘pepper-pot’ (mask) and samples], *mirrors* (planar, circular/elliptical cylinders and toroids), *elements of monochromator* (crystal and grating), and finally a watchpoint. A user can set up a beamline as a sequence of optical elements using a drag-and-drop editor. To add a new element, the user can drag it from the toolbar menu and drop it anywhere in the beamline. The program then pops up a parameter dialog to specify details about the optical element and its exact longitudinal position with respect to a source. Depending on the type of the element, the parameter dialog may have several tabs with basic and advanced parameters.

When the user drags and drops a watchpoint in between the existing elements, after clicking the ‘Save Changes’ button a new Intensity Report for the added watchpoint will appear. Any optical element and watchpoint can be temporarily disabled, as demonstrated by the darker watchpoint element in the beamline definition area of Fig. 2(*b*) ▸. This is done by clicking the ‘PowerOff’ button, which appears when hovering the cursor over the element. Below we will illustrate the definition of the main optical elements currently implemented in *Sirepo*.

### 4.1. Optical element parameters   

#### 4.1.1. Refractive optics and transmission objects   

Transmission objects are typical in Fourier optics. Here, we briefly describe the related optical elements available in *Sirepo*.

A lens is an idealized optical element which has no aberrations and is characterized by focal lengths in the horizontal and vertical planes. It changes the quadratic terms of the phase of the radiation electric field.

**Figure 9:**
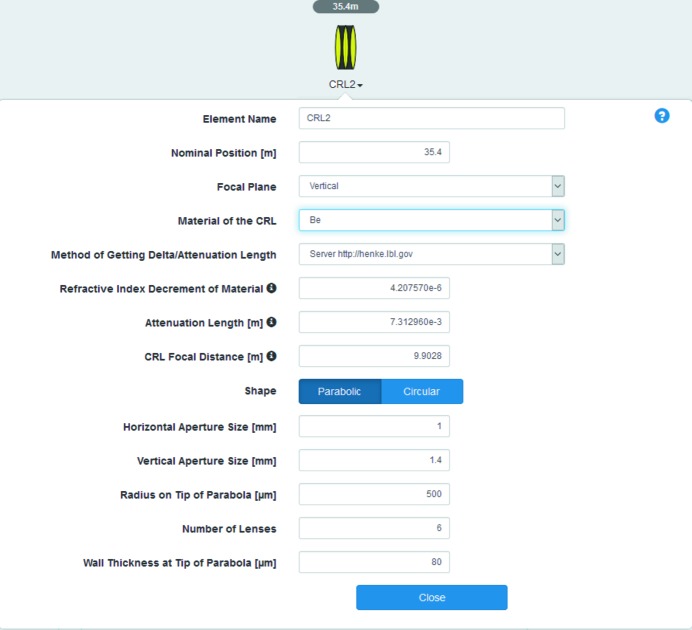
The menu of the CRL.

For a compound refractive lens (CRL) (Snigirev *et al.*, 1996 ▸) element (see Fig. 9 ▸), the values of the refractive index decrement and the attenuation length of the material are dynamically accessible from the Center for X-ray Optics (CXRO) online database (http://henke.lbl.gov/optical_constants), a widely used resource with a comprehensive database related to X-ray interactions with matter. The refractive index decrement and the attenuation length strongly depend on the type of material and the photon energy of interest, therefore the possibility to dynamically query the CXRO database helps users to obtain the parameters automatically. The detailed description of this feature is available on our Wiki (https://github.com/radiasoft/sirepo/wiki). The focal length of the CRL is calculated as well, allowing scientists to more easily estimate the configuration of the CRL for their needs.

The Fiber optical element, which can be used for testing X-ray beam coherence (Kohn *et al.*, 2001 ▸), has been implemented and used in *SRW* for some time (Chubar *et al.*, 2001 ▸, 2013*b* ▸) and has recently been made available in *Sirepo*. The refractive index decrement and the attenuation length parameters are obtained automatically by the same method that is used for the CRL.

The Aperture and Obstacle optical elements can be used separately or together to simulate slits, which are extensively used in beamlines. Users can place rectangular and circular apertures and obstacles, with specified sizes and transverse positions.

The Mask (‘pepper-pot’) element can be used as a wavefront Hartmann sensor for X-rays (Idir *et al.*, 2017 ▸). This element is being developed as part of a collaboration with the Metrology group at Brookhaven National Laboratory (BNL).^1^ We are currently working on improving the Mask implementation before it is available to users.

**Figure 10:**
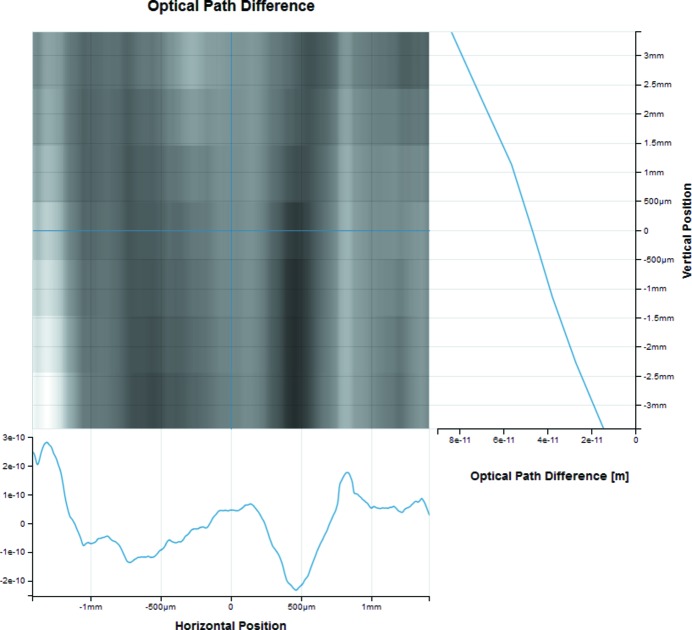
The optical path difference of a 2D mirror height profile due to surface errors, defined as a ‘transmission’ object (modifying only the radiation phase in this case).

For many types of experiments (elastic scattering, transmission microscopy, *etc*.) samples can be represented as transmission objects. More details on the Sample optical element and an example are provided in §4.3. A height profile (*e.g.* of a mirror) can also be represented as a transmission object. Fig. 10 ▸ demonstrates the optical path difference describing the quality of a mirror surface.

#### 4.1.2. Mirrors   

The orientation of mirrors and gratings can be defined *via* grazing angle and/or *via* coordinates of the normal and tangential vectors to the mirror surface at its center point, in the frame of the incident beam. The vector coordinates are updated automatically whenever the grazing angle value is changed by the user.

Planar, circular/elliptical cylindrical and toroidal mirrors are available. In *SRW*, mirror propagators are implemented using the stationary phase method, which allows one to take into account (error-free) surface shape and orientation of the mirror in the frame of the incident beam (Canestrari *et al.*, 2014*b* ▸). Users can also add 1D and 2D height profiles (*e.g.* metrology data) describing imperfections of the mirror surfaces. The optical path difference can be visualized to better understand the surface quality of a mirror as demonstrated in Fig. 10 ▸.

#### 4.1.3. Crystals and gratings   

**Figure 11:**
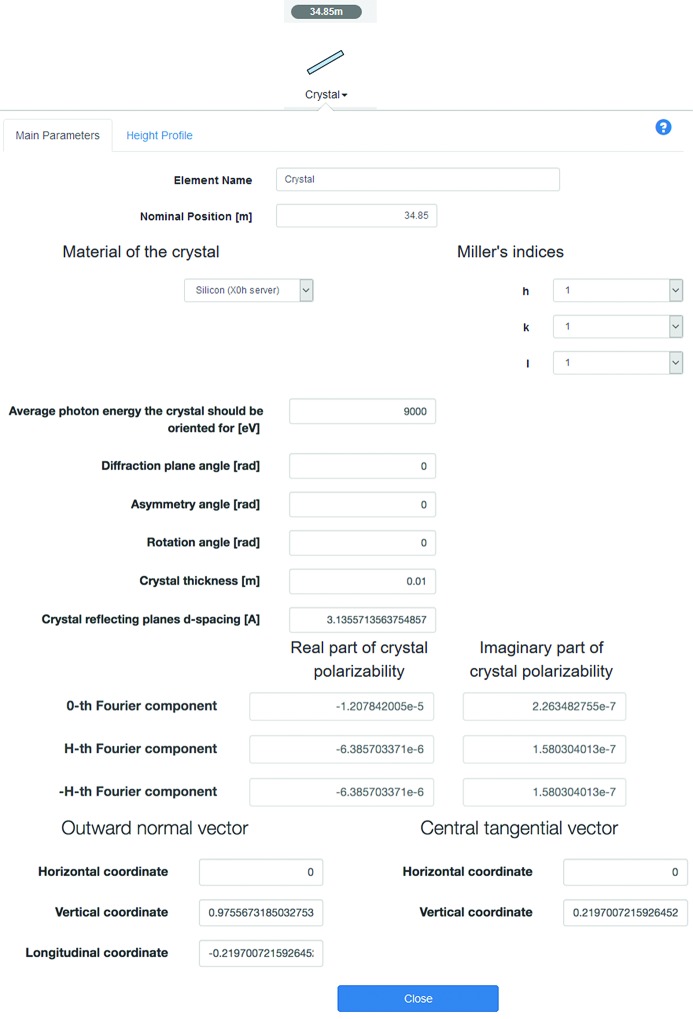
Parameters of the Crystal optical element.

Perfect crystal and grating optical elements are available in *Sirepo* for the simulation of monochromators for hard and soft X-rays. Individual crystals are used as parts of double-crystal monochromators – optical elements, used in most hard X-ray beamlines, in which dynamical diffraction on single crystals is used to cut a narrow bandwidth from a wide spectrum of synchrotron radiation. In *SRW*, the crystal propagator for the radiation electric field is implemented based on the Zachariasen formulae (Zachariasen, 1945 ▸; Sutter *et al.*, 2014 ▸). Options of the propagator for Bragg reflection and transmission geometries are available, while the one for the Laue geometry is still under development. Fig. 11 ▸ depicts the parameters of the Crystal optical element in *Sirepo*.

Based on the specified photon energy, the material and Miller’s indices, the crystal reflecting planes’ *d*-spacing and the real and imaginary parts of the crystal polarizability/susceptibility χ_0_, χ_*h*_ (chi-zero, chi-h) are automatically obtained by programmatically accessing the API provided by the X-ray Server (http://x-server.gmca.aps.anl.gov/x0h.html). Any change in the photon energy, material or Miller’s indices will trigger a new request to the server and the corresponding fields will be populated by the newly received values. Any change of the angles, the *d*-spacing or the components of the crystal polarizability results in a recalculation of the components of the normal and tangential vectors by *Sirepo* to correctly orient the crystal for the particular energy and material, providing a helpful tool for beamline scientists attempting to simulate beamlines with crystal monochromators. Our Wiki (https://github.com/radiasoft/sirepo/wiki) demonstrates the use of this feature.

**Figure 12:**
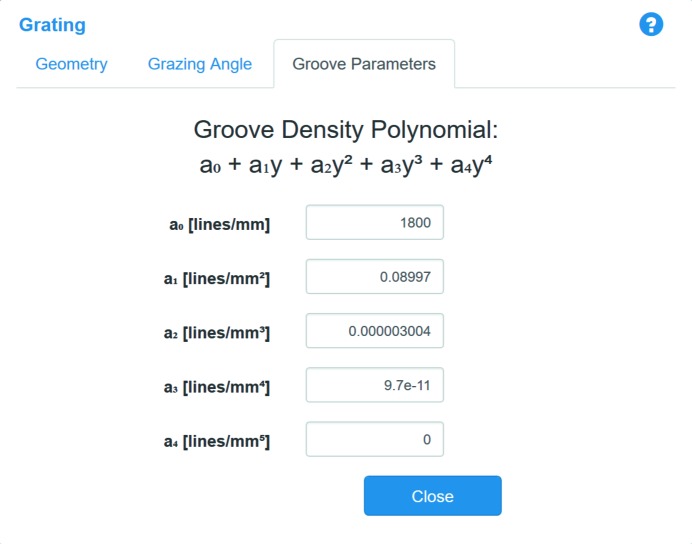
Parameters of the Grating optical element.

Gratings are often used to generate angular dispersion in a polychromatic incident soft X-ray beam, which allows for the creation of an efficient monochromator, *e.g.* by installing a slit at some distance after the grating. Calculation of the wavefront propagation through gratings in *SRW* also utilizes the stationary phase (or the ‘local ray-tracing’) method (Canestrari *et al.*, 2014*a* ▸). Parameters of the grating element can be configured *via* its menu (Fig. 12 ▸). The grating may be defined to have a variable line/groove spacing, with the groove density as a function of longitudinal position defined by a fourth-order polynomial.

### 4.2. Simulation example: propagation of radiation from source to sample   

Along with some textbook examples, *Sirepo* implements a number of predefined virtual beamlines: the CHX, HXN, SRX, FMX, SMI and ESM beamlines of NSLS-II (https://www.bnl.gov/ps/beamlines) and the SXR beamline of LCLS at SLAC (https://lcls.slac.stanford.edu/instruments).

**Figure 13:**
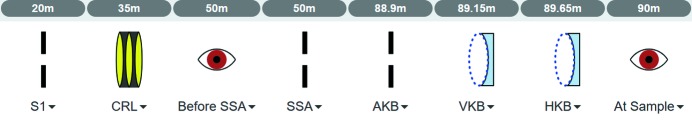
Definition of a simplified hypothetical beamline of the simulation example in *Sirepo*/*SRW*.

The following text covers several levels of simulations which *Sirepo* allows to perform using a simplified hypothetical beamline. Fig. 13 ▸ shows the beamline layout as defined *via* *Sirepo*. Each watchpoint element produces a separate intensity report at the distance where the watchpoint is introduced to the beamline. We will pay attention to the initial intensity report (at 20 m from the source) and the intensity reports corresponding to the watchpoints ‘Before SSA’ (50 m from the source) and ‘At Sample’ (90 m from the source) in all simulations described below.

The beamline example uses the idealized undulator source. We tuned the undulator to produce the fundamental harmonic at 1.6 keV with the ‘NSLS-II Low Beta Day 1’ predefined electron beam parameters (see Fig. 4 ▸ and Table 1 ▸), and for our simulation we have selected the fifth undulator radiation harmonic with the photon energy of 8 keV. This photon energy value and the longitudinal position of the first optical element can be defined in a dialog activated through the ‘Initial Wavefront’ button. For simplicity, the monochromator is not included in these simulations, and the radiation is assumed to be perfectly monochromatic.

The rectangular aperture associated with beam slit ‘S1’ is located 20 m downstream from the source, and has the size of 1 mm × 1 mm (h × v). To focus the beam at the Secondary Source Aperture (SSA), located 50 m from the source, we employ a beryllium CRL. The SSA size is 15 µm × 10 µm (h × v). A watchpoint ‘Before SSA’ is used to observe the intermediate intensity distribution as a wavefront propagates through the preceding optical elements. The second part of the beamline (downstream from the SSA) is designed to utilize a pair of elliptical mirrors in Kirkpatrick–Baez geometry (Kirkpatrick & Baez, 1948 ▸) to produce a sub-micrometre X-ray beam spot at a sample position ∼90 m from the source. The vertically focusing mirror ‘VKB’ is located at a distance of 89.15 m from the source, while the horizontally focusing mirror ‘HKB’ is at 89.65 m. Both mirrors have a grazing incidence angle of 3.6 mrad. This system allows for focusing the beam to a size of ∼100 nm in the horizontal and vertical planes at the sample.

**Figure 14:**
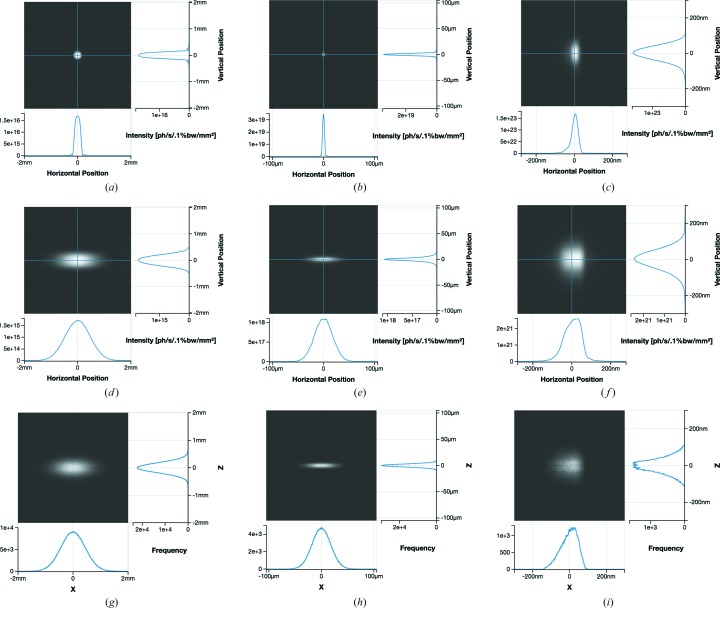
Initial intensity report (at 20 m) and intensity reports for the watchpoints ‘Before SSA’ (at 50 m) and ‘At Sample’ (at 90 m) (left to right) in *Sirepo*. The first row corresponds to the single-electron (fully coherent) *SRW* simulation, the second to the multi-electron (partially coherent) *SRW* simulation, and the third to the fully incoherent *SHADOW3* simulation.

Here, we guide the reader through fully and partially coherent *SRW* simulations of the beamline. We also present fully incoherent simulations of the same beamline, using the geometrical ray-tracing approach implemented in the *SHADOW3* code, which is also available in *Sirepo*. Fig. 14 ▸ displays the intensity distributions at three locations: 20 m (initial intensity), 50 m (‘Before SSA’) and 90 m (‘At Sample’) from the source, resulting from different types of calculations. The first row corresponds to a single-electron (fully coherent) *SRW* simulation. The second row corresponds to a multi-electron (partially coherent) *SRW* simulation. The third row corresponds to a fully incoherent *SHADOW3* simulation.

The single-electron simulation is usually finished within a few minutes. By default, *Sirepo* performs these fast single-electron simulations; however, if the user wants to perform a more realistic multi-electron simulation, taking into account the distribution of electrons over the phase-space volume of the emitting electron beam, then the ‘Partially Coherent’ tab on the Beamline page can be used. These partially coherent simulations require more computing resources, and can run in parallel from a few minutes to a few days, depending on the parameters of the simulation and available resources. The corresponding results for our test case can be observed in Fig. 14 ▸ (second row). The intensity plot is interactive and is updated periodically to show the latest results. The presented intensity distributions were obtained after about 30 min of execution using 21 cores of a multi-processor system, converging after averaging of about 5000 macro-electrons. The corresponding *SRW* simulation example can be found in *Sirepo* (https://sirepo.com/srw#/beamline/LA5qG1J1).

The equivalent *SHADOW3* beamline was recreated in *Sirepo* (https://sirepo.com/shadow#/beamline/wsGlwMqv). The resulting initial intensity distribution and intensity distributions ‘Before SSA’ and ‘At Sample’ are displayed in Fig. 14 ▸ (third row). This ray-tracing calculation lasted a few minutes. It can be seen that the results are comparable with ones obtained from *SRW* partially coherent simulations. This is also illustrated by Table 2 ▸, where the spot sizes obtained by the different simulations are listed. The relatively good agreement between the partially coherent calculations with *SRW* and the incoherent *SHADOW3* calculations are explained by a relatively low degree of coherence of the radiation in this case, in particular in the horizontal plane, where the intensity distributions are mainly dominated by the contributions from the finite-emittance electron beam.

The absence of significant slit diffraction in this example makes the results of the partially coherent and incoherent simulations relatively close, even in the vertical plane, where the degree of the radiation coherence is relatively high. The existing differences between some spot dimensions are in part due to the use of an incoherent Gaussian beam as the source for the *SHADOW3* simulations, rather than starting with undulator radiation. A more detailed comparison between results of partially coherent simulations with *SRW* and incoherent simulations with *SHADOW3*, for different optical schemes, with different degrees of source coherence and different contributions from slit diffraction, has been previously presented by Canestrari *et al.* (2014*b* ▸).

### 4.3. Simulation of X-ray scattering from experimental samples   

Simulation of radiation scattering by experimental samples is important for experiments at light source facilities (Chubar *et al.*, 2017 ▸).

**Figure 15:**
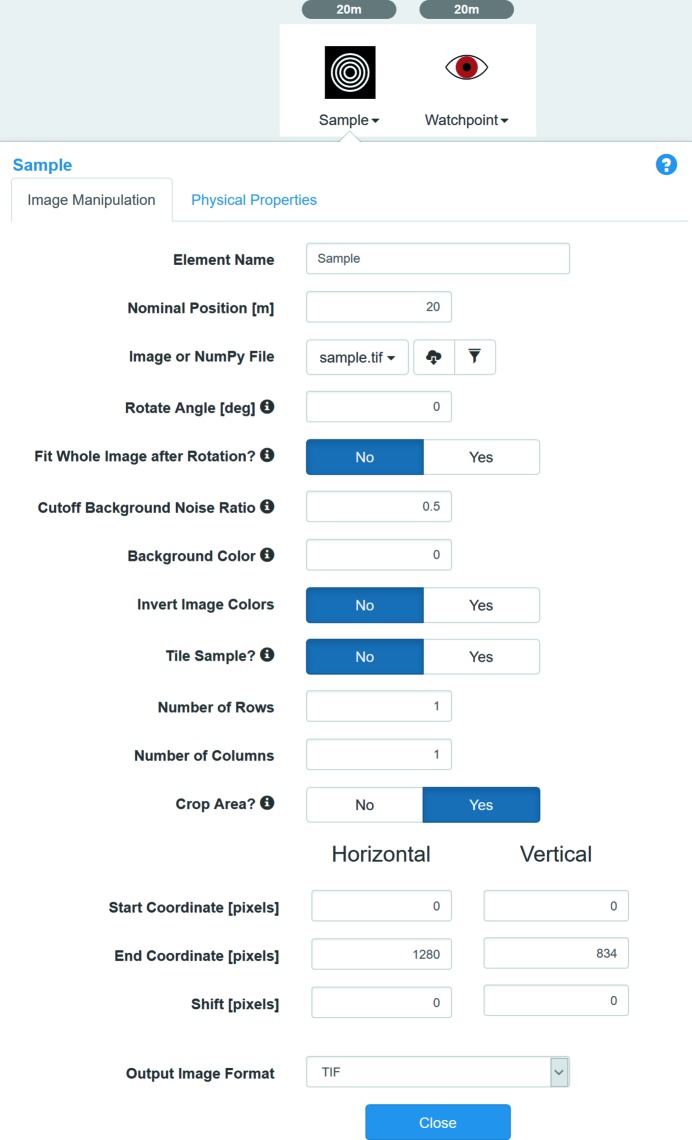
Parameters of the Sample optical element.

In collaboration with the Center for Functional Nanomaterials (CFN) of BNL, we started implementation of a Sample library in *SRW* and created the corresponding Sample optical element in *Sirepo* (Fig. 15 ▸). Users can provide an electron microscope image or a NumPy file describing a real or virtual experimental sample, together with specified spatial resolution of the image and thickness of the sample. The material of the sample can be specified in a drop-down menu, and the refractive index decrement and attenuation length of the material can be obtained automatically from the above-mentioned CXRO online database, the same way it is done for the CRL (see §4.1.1). Positions of the sample can be specified relative to the source and the origin of the incident beam frame.

The Sample library accepts many types of input, *i.e.* image files in different formats (TIFF, PNG, JPEG, BMP, *etc.*) or a NumPy array file with the data of the sample image.

Python functions have been developed to implement these new capabilities *via* the Python API of *SRW* and *Sirepo*. The Sample image is read by means of the Python Image Library (https://pillow.readthedocs.io) and is converted to a NumPy array. The area of interest can then be cropped from the original image by means of the parameters (see menu in Fig. 15 ▸). Next, a function creates an *SRW* transmission object treating the thickness of the material at each pixel proportional to the gray level of the pixel. The refractive index decrement and attenuation length parameters are used along with the thickness of the material in the amplitude transmission and optical path difference calculations for each pixel. Finally, the resulting transmission object is used by *SRW* to perform the wavefront propagation through the element and a following drift space to obtain a diffraction pattern at the specified distance from the sample.

**Figure 16:**
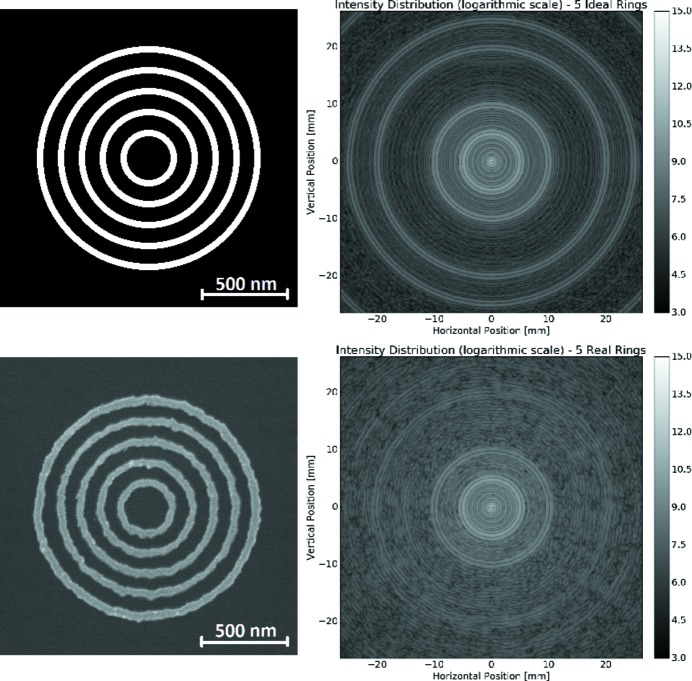
Concentric rings sample (left) and simulated diffraction pattern created by it as observed at 4.81 m (right): ‘ideal’ fabrication error-free case (upper image plots) and the case of a sample object generated for simulations from a real nano-fabricated sample (lower image plots). The outermost diameter/size of both samples is ∼1.35 µm. The simulations were performed with the NSLS-II CHX beamline layout.

Fig. 16 ▸ shows the scattering pattern after the coherent (9.65 keV) radiation propagation through nano-object samples manufactured at CFN (Lhermitte *et al.*, 2017 ▸). In the figure, input images of both the real sample and the ‘ideal’ sample of the same topology (*i.e.* concentric rings) are presented together with their corresponding diffraction patterns. The patterns are similar in the two cases, except that the one corresponding to the real sample contains more ‘noise’ (speckles). This is presumably due to the existing imperfections of the real sample. Analysis of the accuracy of such diffraction images and speckle patterns is a subject of ongoing work. The Sample library will be further developed and expanded, so that it can be used in simulations of coherent scattering and microscopy experiments at light source facilities.

### 4.4. Application of *Sirepo*/*SRW* at NSLS-II   

*Sirepo* and *SRW* have been intensively utilized at the NSLS-II light source facility in many aspects. For instance, spectrum-based alignment of in-vacuum undulators (IVUs) was guided by realistic *SRW* calculations of on-axis undulator radiation spectra for SRX, CHX, LIX and SMI beamlines (Chubar *et al.*, 2018 ▸). Another example is a study of the coherence properties in the hard X-ray regime at the CHX beamline. Based on the commissioning results, a realistic simulation of the whole beamline, including source and optics, has been performed leveraging a ‘virtual beamline’ module of *SRW* code in *Sirepo* (Chubar *et al.*, 2017 ▸; Wiegart *et al.*, 2017 ▸, 2018 ▸). This virtual beamline capability was used to simulate partially coherent small-angle scattering patterns of samples relevant to the CHX science case, mimicking ‘detector images’ of real experiments. Such source-to-detector simulations of experiments allow for an accurate estimation of feasibility of a given experiment, for an optimization of the beamline setup and interpretation of experimental results.

## 5. Performance overhead benchmarking   

In order to optimize the *Sirepo* interface for *SRW* and to minimize the total waiting time for simulations, we collected data regarding the differences between *SRW* run times when using the GUI as compared with the command line interface (*i.e.* Python scripts). We also measured the distribution of total user waiting time between different processes, such as calculation, data preparation and data transfer to a client. Efficiencies of three different servers running *SRW* were tested as well. It was discovered that for nearly all tested simulations the user waiting time with the GUI was greater, with some simulations taking up to three times longer. The difference was often the result of time spent on data transfer.

First, we measured total user waiting times for single-electron emission and wavefront propagation simulations *via* the GUI and the command line. The waiting times were measured for three different cases of radiation wavefront sampling, *i.e.* resolution, to see whether this affected the GUI and command line performance differently.

**Figure 17:**
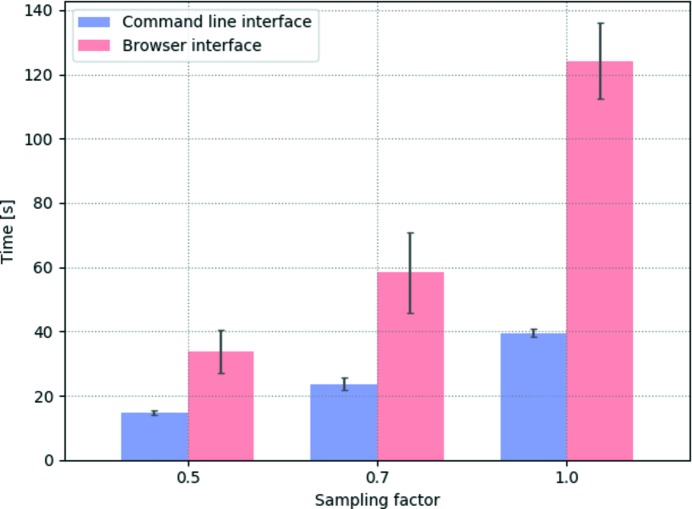
Total user waiting time *versus* sampling factor of the initial undulator radiation wavefront in the NSLS-II FMX beamline single-electron simulation performed on the NSLS-II Vagrant server.

The tests have shown that with the increase in resolution the GUI performance lagged further behind the command line performance. With the increase in resolution, the amount of calculation that is required should reasonably increase by equal factors, regardless of the interface used. However, a browser-based GUI must transfer all data from a server, which contributes to the waiting time, especially for large data sets. With the increase in resolution, more data need to be transferred from server to client, increasing the latency. Fig. 17 ▸ illustrates a series of data that were collected and averaged; the vertical bars show the standard error.

**Figure 18:**
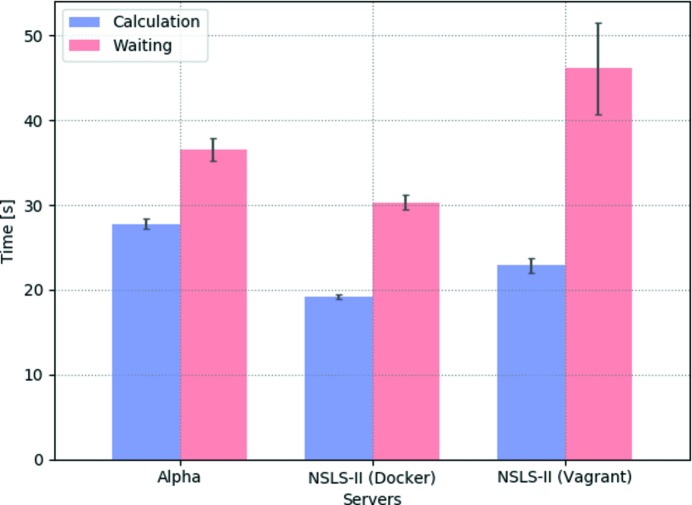
Comparison of the calculation time and waiting time for three different servers (each simulating the NSLS-II FMX beamline with *SRW*, using a sampling factor of 0.7): RadiaSoft’s Alpha server, and two installations of *Sirepo* at NSLS-II, one using Docker and the other using the VirtualBox virtual machine provided *via* a Vagrant container.

To quantify how the data transfer time affected the performance of the GUI, we compared the calculation time and the total waiting time for three servers running the same *SRW* calculation (Fig. 18 ▸).

The ‘Alpha’ server is a single Linux node from a commercial cloud computing provider, with the *Sirepo* back-end running inside Docker. The NSLS-II Docker server is a single blade running Debian Linux, with 36 cores in two sockets. Here again the *Sirepo* back-end was running inside Docker. The NSLS-II Vagrant server is a standalone host system running MS Windows with 28 cores, half of which are allocated to a VirtualBox VM running *Sirepo* services. From Fig. 18 ▸ we see that the data transfer contributes 25–50% of the total user waiting time.

Different portions of time spent for data transfer can be explained by the different server configurations and different virtualization technologies. For instance, the NSLS-II Vagrant server was found to spend about half of the user waiting time on data transfer, which can be explained by larger overhead from the guest operating system within the VirtualBox virtualization environment, in contrast to more efficient containerization *via* Docker technology, utilized on other NSLS-II servers and the Alpha. To optimize *Sirepo* performance, the amount of transferred data must be minimized.

**Figure 19:**
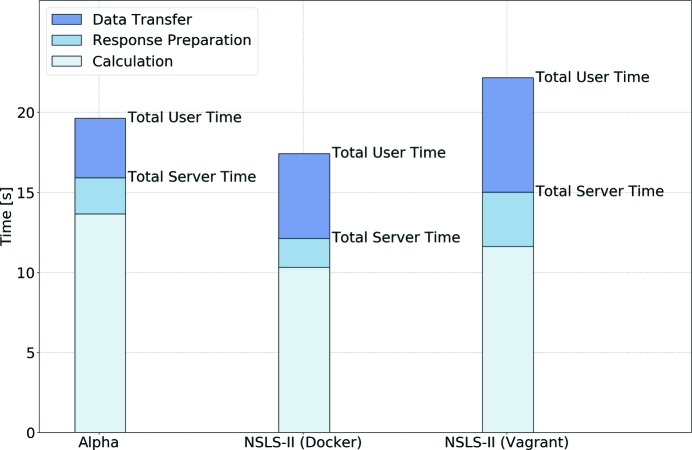
Components of the total user waiting time. Total server time is the aggregate value of the calculation time and the response preparation time. Total user time is the aggregate value of the total server time and time for data transfer. The data were gathered on simulations for the NSLS-II FMX beamline case with a sampling factor of 0.5.

Tests were also performed with *Sirepo* to measure the time spent by the server on preparation of the results before sending them to the client (browser), such as conversion of *SRW* calculation results to JSON format. This was done by subtracting the calculation time from the total server time. Fig. 19 ▸ illustrates the results: it is clear that the longest time in the considered single-electron simulation case is the calculation. As expected, tests show that the calculation time *via* the command line interface is almost identical to the calculation time of *SRW* in *Sirepo*, meaning that the overhead of the virtual machines is minimal.

The response preparation was found to be reasonable, as compared with the *SRW* calculation time. It does not exceed 15% of the total waiting time. The time spent on data transfer between the *Sirepo* server and the browser-based GUI represents the most significant performance overhead compared with command line execution. However, it is expected behavior for a distributed system: a browser-based GUI (client) must wait until the results are transmitted over the network.

**Figure 20:**
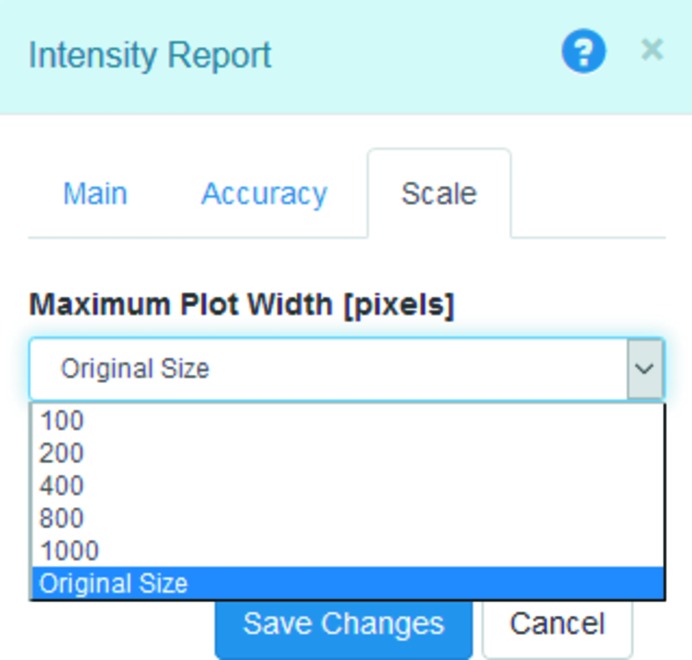
A menu in the *Sirepo* interface for limiting the size of the resulting image to be sent to client

This issue has recently been addressed in *Sirepo* by means of limiting the maximum image size being passed from server to client (see Fig. 20 ▸). This is done by resizing the original data set produced by *SRW*, using the 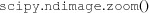 function, when the width of the image is greater than the specified amount of pixels (original size is the default value). The feature was tested on large data files corresponding to up to 10000 × 10000 pixel images, and showed a considerable reduction in data transfer time.

The profiling tests described above involved relatively fast, single-electron fully coherent *SRW* calculations. In the case of multi-electron partially coherent calculations, delays due to the data transfer and other factors relative to the use of *Sirepo* are smaller compared with the calculations themselves, even if periodic updates of plots are sent to the corresponding reports. The multi-electron partially coherent calculations may be substantially speeded up *via* parallel execution. The above considerations suggest that both *Sirepo* and command line (Python) interfaces to *SRW* would benefit equally from this speed-up, as it is entirely on the server side. In *Sirepo*, such calculations are decoupled from the visualization procedure, and therefore the speed of the calculation is not affected by the nature of the distributed system.

Benchmarking of both *Sirepo* and *SRW* is an important topic. As the development is ongoing, we will continue improving the code and benchmarking the results.

## 6. Distribution of *Sirepo*   

The software developed for this project has been released under an open source license and published on GitHub (https://github.com/radiasoft/sirepo) and other repository sites. As an out-of-the-box application, *Sirepo* can be obtained within a container, either Docker (https://hub.docker.com/r/radiasoft/sirepo) or Vagrant (https://app.vagrantup.com/radiasoft/boxes/sirepo).

Docker is an open platform for distributed applications. It enables rapid deployment of applications to the cloud. Using Docker on Linux, it is possible to create a file that contains scientific code(s), plus all required tools and dependencies, which can then be copied to any Linux server or cluster and rapidly activated. A user can connect to the container *via* console interface, or the software can be accessed remotely through a web-based user interface. This removes the ‘pain’ of multi-component software installation on Linux, and enables all the advantages of cloud-based scientific computing by providing on-demand access *via* a local cluster, supercomputer or commercial cloud provider. Docker-compatible execution environments are available at many national supercomputing centers.

Vagrant enables cross-platform containerization of applications. Just like Docker, it is used to create and configure lightweight, reproducible and portable Linux development environments. This is essential for developing and testing Linux applications on non-Linux computers.

Since our source code resides on GitHub, it provides a way to efficiently test the code and distribute it *via* the Python Package Index (PyPI) and the Docker repositories. This process is managed using Travis CI, a continuous integration and continuous delivery platform. After each commit to the GitHub repository and successful tests in the Travis CI environment, a new version of the Python-installable package is uploaded to the PyPI server and a new container is generated and placed automatically in the Docker repository.

*SRW* and its Python interface, fully compatible with a given version of *Sirepo*, is included in the *Sirepo* distribution container. The most recent cross-platform canonical version of *SRW* for Python can also be downloaded from a dedicated *SRW* GitHub repository (https://github.com/ochubar/SRW); however, this version is not guaranteed to be fully supported by *Sirepo*.

## 7. Summary   

We have presented *Sirepo*, an open source cloud-computing framework, which includes a sophisticated browser-based GUI for X-ray optics simulations. Currently, *Sirepo* is interfaced with popular codes in the fields of synchrotron radiation source and optics simulations, such as *SRW* and *SHADOW3*, and particle accelerators (*Elegant*, *Hellweg* and *Warp*). *Sirepo* is a flexible framework that can be relatively easily integrated with scientific codes to provide a convenient GUI for simulations in the cloud.

We described in detail the features of the *Sirepo* interface for *SRW*, a program for physical optics simulations. *Sirepo* for *SRW* currently contains predefined textbook examples as well as simulations of the wavefront propagation through existing beamlines at NSLS-II and LCLS. Users benefit from both Source and Beamline simulation pages. In the Source page, users can simulate and optimize the source of the synchrotron radiation (*e.g.* undulator, dipole, *etc.*). In the Beamline page, one can construct a ‘virtual’ beamline emulating the layout of real X-ray or general optical beamlines.

*Sirepo* utilizes interactive widgets and dynamically accessed data from community databases for X-ray optics. Based on benchmarking tests, we have worked to ensure reliability and to minimize overheads related to the use of *Sirepo* with large datasets. *Sirepo* can have a number of important applications for light source facilities and in other areas.

## Figures and Tables

**Table 1:** Electron beam parameters of the ‘NSLS-II Low Beta Day 1’ predefined beam

Parameter	Value(s)	
Energy (GeV)	3	
Current (A)	0.5	
RMS energy spread	0.89 × 10^−3^	

	Horizontal	Vertical
Emittance	0.9 nm	8 pm
Beta-function	1.84	1.17
RMS size (µm)	40.69	3.06
RMS divergence (µrad)	22.12	2.61

**Table 2:** Comparison of the beam spot sizes (horizontal and vertical FWHM) at different distances (20, 50 and 90 m) from the source for different calculation methods

	At 20 m (µm)		Before SSA (50 m) (µm)		At sample (90 m) (nm)	
Calculation method	Horizontal	Vertical	Horizontal	Vertical	Horizontal	Vertical
*SRW* (single-electron)	253.6	262.3	4.7	4.5	36.6	92.4
*SRW* (multi-electron)	1107.0	464.2	42.4	6.0	106.6	118.7
*SHADOW3*	1032.6	503.8	41.6	6.7	106.4	80.3

## Footnotes

1 DOE FWP grant DE-SC0012704.
